# Health-related quality of life and related factors of military police officers

**DOI:** 10.1186/1477-7525-12-60

**Published:** 2014-04-27

**Authors:** Franciele Cascaes da Silva, Salma Stéphany Soleman Hernandez, Beatriz Angélica Valdivia Arancibia, Thiago Luis da Silva Castro, Paulo José Barbosa Gutierres Filho, Rudney da Silva

**Affiliations:** 1 University of State of Santa Catarina, Adapted Physical Activity Laboratory, Pascoal Simone Street, 358, 88080-350 Coqueiros, Florianopolis, SC, Brazil; 2 Physical Education College, University of Brasilia, Campus Darcy Ribeiro, Brasilia, CEP 70910-900, Brazil

**Keywords:** Body composition, Coronary disease, Quality of Life, Police

## Abstract

**Purpose:**

The present study aimed to determine the effect of demographic characteristics, occupation, anthropometric indices, and leisure-time physical activity levels on coronary risk and health-related quality of life among military police officers from the State of Santa Catarina, Brazil.

**Methods:**

The sample included 165 military police officers who fulfilled the study’s inclusion criteria. The International Physical Activity Questionnaire and the Short Form Health Survey were used, in addition to a spreadsheet of socio-demographic, occupational and anthropometric data. Statistical analyses were performed using descriptive analysis followed by Spearman Correlation and multiple linear regression analysis using the backward method.

**Results:**

The waist-to-height ratio was identified as a risk factor low health-related quality of life. In addition, the conicity index, fat percentage, years of service in the military police, minutes of work per day and leisure-time physical activity levels were identified as risk factors for coronary disease among police officers.

**Conclusions:**

These findings suggest that the Military Police Department should adopt an institutional policy that allows police officers to practice regular physical activity in order to maintain and improve their physical fitness, health, job performance, and quality of life.

## Abstract (pt)

**Resumo:**

**Objetivo**

O presente estudo teve como objetivo determinar o efeito das características demográficas, ocupacionais, antropométricas e dos níveis de atividade física no lazer sobre o risco coronariano e qualidade de vida relacionada à saúde de policiais militares do Estado de Santa Catarina, Brasil.

**Métodos**

A amostra foi composta por 165 policiais militares que preencheram os critérios de inclusão do estudo. Foram aplicados os questionários *International Physical Activity Questionnaire*, *Form Health Survey-36* e Inventário de Risco Coronariano, além de uma planilha de dados sócio-demográficos, ocupacionais e antropométricos. As análises estatísticas consistiram em análise descritiva seguida de correlação de *Spearman* e análise de regressão linear múltipla utilizando o método *backward*.

**Resultados**

A relação cintura estatura foi identificada como fator de risco para uma baixa qualidade de vida relacionada à saúde. Além disso, o índice de conicidade, percentual de gordura, tempo de serviço na Polícia Militar, minutos de trabalhos por dia e níveis de atividade física no lazer foram identificados como fatores de risco para doença coronariana entre os policiais.

**Conclusões**

Estes resultados sugerem que os Departamentos de Polícia Militar devem adotar uma política institucional que viabilize a prática da atividade física regular, com o objetivo de manter e aperfeiçoar as aptidões físicas, com vistas à melhoria de sua saúde e, por conseguinte para o seu aprimoramento técnico profissional e sua qualidade de vida.

## Introduction

Although the field of public security sets high prerequisite entry standards for physical fitness and good health, the demands of police work do not allow police officers to maintain their physical fitness, which tends to deteriorate over time [1,2].

This decline in physical fitness among police officers is primarily due to a general decrease in physical activity throughout their careers. A consequence of decreased physical activity is increased body mass and increased risk of developing health problems [1,3].

Previous studies conducted with police officers have found that higher anthropometric indices such as body mass index, waist circumference, abdominal circumference, body fat and total body mass are related to years of police service. Such studies suggest that improving physical fitness is essential to improve police officers’ performance and quality of life [1-8].

There are also social and psychological demands related to police work, such as the pace of daily work, occupational responsibilities, and distressing situations, as well as factors such as poor diet and physical inactivity that can initiate health problems and affect quality of life among police officers. Therefore, the aim of this study was to determine the effect of demographic characteristics, occupation, anthropometric indices, and leisure-time physical activity levels on police officers coronary disease risk and quality of life.

## Methods

This descriptive correlational study was submitted for consideration and approved by the Ethics Committee on Research involving Human Subjects of the University of the State of Santa Catarina, under protocol number 130/2011, according to resolution 466/2012 of the Ministry of Health of Brazil with regard to the Declaration of Helsinki and its subsequent.

The study sample was selected from active-duty police officers from the Military Police Battalion of Santa Catarina, located the State of Santa Catarina, Brazil (N = 224). The sample was composed of military police officers who agreed participate in the study, were older than 18 years and were active duty. Policemen who retired because of illness and those who did not fully complete questionnaires were excluded from the sample. Furthermore, to avoid possible bias, women were excluded from the analysis because there were only 11 female police officers.

The sample size calculation was conducted using *SampleXS for Windows,* adopting a 50% obesity prevalence, a margin of error less than 5%, and a design effect of 1.5 points. A sample size calculation for finite populations determined a need for a random sample of 187 research subjects. In total, 208 police officers consented to participate. However, 32 subjects were excluded for not fully completing questionnaires e 11 female subjects, leaving a final sample of 165 subjects (n).

Subjects were interviewed to collect demographic and occupational data (gender, age, degree official duties, minutes of work per day, workdays per week, years of service) and anthropometric indices (weight, height, waist circumference and fat percentage). They also completed three questionnaires: a) the *International Physical Activity Questionnaire* long version (IPAQ) [9]; b) *Short Form Health Survey* (SF-36) [10]; and c) the Coronary Risk Inventory, created by the *Michigan Heart Association*.

The body composition assessment was performed using anthropometric techniques developed by Lohman, Roche, and Martorell [11]. Body mass index was measured using a Cuori® CUO9340 digital scale, body height was verified with a Filizola® digital stadiometer, and waist circumference was measured with a Cescorf® metric tapeline. Using these data, the conicity index (CI) and waist-to-height ratio (WHtR) for each subject were determined.

The CI is an indicator of obesity and body fat distribution that is associated with cardiovascular risk factors and is calculated with the following equation: CI = WC/0,109. BW/BH, where CI = Conicity Index, WC = Waist circumference (m), BW = Body Weight (kg), and BH 107 = Body Height (m) [12,13]. WC is an indicator used in measuring centralized tissue distribution and is more strongly associated with levels of visceral abdominal adipose tissue than waist-to-hip ratio (WHR) [14]. The average CI value established for men was reported as 1.23 and for women as 1.18 [13]. The average WC value for established for men was reported 88 cm and for women as 83 cm [12].

WHtR is strongly associated with several cardiovascular risk factors and is calculated with the following equation: WHtR = WC/BH, where WHtR = waist-to-height ratio, WC = waist circumference (cm) and BH = body height (cm). The reference values of 0.52 for men and 0.53 for women were used [15].

Measurement of skinfold thickness was performed using a Cescorf® plicometer using the Jackson and Pollock three-site skinfold test (chest, abdomen and thigh for men, triceps, suprailiac and thigh for women). The measurements were performed three times, and the average value of the three assessments was recorded.

The IPAQ consists of 27 questions that are used to evaluate a subject’s estimated metabolic equivalent (MET) based on 5 domains of physical activity: 1) work, 2) domestic tasks, 3) transportation, 4) leisure/sport/recreation, and 5) time spent sitting [9]. A subject’s physical activity level was classified as low (< 600 Mets-min/week), moderate (600 to 1500 Mets-min/week), high (1500 to 3000 Mets-min/week) or very high (> 3000 Mets-min/week). Subjects were further classified as sedentary (< 600 Mets-min/week), active (1500 to 3000 Mets-min/week) or very active (> 3000 Mets-min/week). This study only examined the leisure-time domain in a typical week related to recreation, sport, exercise and leisure activities. This domain are assessed how many days per week and how much time per day the subject performs for at least 10 continuous minutes of walking, vigorous physical activity (such as running, swimming fast, quick, weight cycling, among others) and moderate activity (pedaling pace moderate, swimming, gymnastics and dance, among others).

The SF-36 is a generic instrument used to assess health-related quality of life related to health. The questionnaire comprises 36 items that correspond to eight domains grouped into two components: 1) Physical component summary: functional capacity, general state of health, pain and physical aspects and 2) Mental component summary: mental health, vitality, social aspects and emotional aspects [10]. Each item of the SF-36 is assessed using points scored on a Likert scale, using the criteria and formula proposed by Ware and Gandek [10]. Final scores range from 0 (worst quality of life) to 100 points (best quality of life). Domain scores are derived from the questions related to them.

The Coronary Risk Inventory proposed by the *Michigan Heart Association* is a scale that assesses eight risk factors related to body weight, smoking, blood pressure, sex, age, physical activity profession, and family history. Each risk factor has six response options, which are associated with a point value. The sum of points obtained represents a subject’s relative risk of developing coronary disease [16].

Data analysis was performed using Microsoft Excel 2010, and statistical analyses were performed with *Statistical Package Social Science* (SPSS) 20.0. The data were summarized by descriptive statistics (mean, standard deviation, simple frequency, percentage and range). A confidence interval of 95% (CI 95%) was adopted and a significance level of *p* < 0,05 was used for all analyses.

Given the distribution of the data shown by the Kolmogorov-Smirnov test, the comparison of the physical component of health-related quality of life and overall coronary risk score between subjects by gender was tested by the U Mann–Whitney and associations between demographics, occupation, health status, leisure-time physical activity levels, anthropometric indicators of obesity and quality of life were obtained by Spearman correlation. The data that were correlated and demonstrated residual normality (years of service, minutes of work/day, overall coronary risk score coronary risk, conicity index, waist circumference, waist and height, fat percentage, and leisure-time physical activity) were inserted into a multiple linear regression.

The multiple linear regression model using the backward method for two outcome variables. The first outcome variable was the physical component of quality of life related to health and explanatory variables were years of service, minutes of work per day, workdays per week, overall coronary risk score, conicity index, waist circumference, and waist-to-height ratio, fat percentage, and leisure-time physical activity. The second outcome variable was the overall score of coronary risk and explanatory variables were years of service, minutes of work per day, workdays per week, conicity index, waist circumference, and waist-to-height ratio, fat percentage, physical component of quality of life related health and leisure-time physical activity.

The choice of backward method was based on routines that incorporate all the variables and then by steps, eliminate or not each of them. The backward method executes F statistics for each explanatory variable The lowest value of the partial F statistics is compared with the critical F, F_out_, calculated for a given α critical value. If the lowest value found is less than F_out_, is removed from the model the covariate responsible for the lower value of the partial F statistic. Then, it adjusts the model again with p-1 variables. The partial F statistics are calculated for this model and the process is repeated. Finally, the elimination algorithm terminates when the lowest partial F statistic is not less than F_out_. Variables that showed an F value less than 0.10 were imputed and those who had an F value greater than or equal to 0.10 were removed from the model. The composition of the final model was obtained from the analysis of the results showed that the variables with p less than 0.05.

## Results

The 165 military police officers who participated in this study had a mean age of 37.27 ± 8.099 years with a range from 22 to 54 years of age. They worked an average of approximately 4 ± 1.172 days per week and 809.15 ± 378.597 minutes per day. Table 1 displays their characteristics, including official graduation date, years of service, leisure-time physical activity level and coronary risk classification.

**Table 1:** Characteristics of 165 police officers participated in the study

**Variable**	**n**	**%**
Official graduation		
Soldier	99	60
Corporal	38	23
Sargent	17	10.3
Sublieutenant	2	1.2
Lieutenant	5	3
Capitan	2	1.2
Major	2	1.2
Total	165	100
Operation		
Predominantly administrative	28	17
Predominantly operational	137	83
Total	165	100
Years of service		
1-10	67	40.6
11-20	34	20.6
21-30	62	37.6
Above 31	2	1.2
Total	165	100
Leisure physical activity level		
Low	86	52.1
Moderate	37	22.4
High	24	14.52
Very high	18	10.9
Total	165	100
Coronary risk rating		
Risk much lower than average	10	6.1
Risk lower than average	58	35.2
Medium risk	70	42.4
Moderate risk	25	15.2
High risk	2	1.2
Total	165	100

n, Frequency; %, Percentage.

A general analysis of these data showed that 60% were soldiers. Subjects predominantly worked in the operational sector (83%), had served between 1–10 years (40.6%), had low leisure time physical activity levels (52.1%) and possessed medium risk for developing coronary diseases (42.4%).

In relation the comparison of the physical component of health-related quality of life and overall coronary risk score between subjects according to sex, the Mann–Whitney test revealed a significant difference between men and women for the overall coronary risk score (U = 234.50, p > 0.001). Thus, women were excluded from analysis to avoid possible bias because the subjects were not in the same condition.

*Spearman* correlation analysis demonstrated relationships between demographic characteristics, occupation, leisure-time physical activity levels, anthropometric indicators of obesity, coronary risk, and physical health-related quality of life among the police officers in this study. Table 2, below, illustrates only the significant relationships found in this study.

**Table 2:** Spearman correlation coefficient (Rho) and significance (p) obtained between demographic, occupational, anthropometric, coronary risk and the physical domain variables

**Variable**	**Years of service**	**Days work/week**	**CI (Kg/m**^**2**^**)**	**WC (cm)**	**WHtR (cm)**	**% Fat mass**	**Coronary risk (points)**	**Leisure physical activity level (mets)**	**Physical component (points)**
	**Rho (p)**	**Rho (p)**	**Rho (p)**	**Rho (p)**	**Rho (p)**	**Rho (p)**	**Rho (p)**	**Rho (p)**	**Rho (p)**
Age (years)	0,944***		0,513***	0,239**	0,455***		0.397***		−0,315***
Years of Service			0,479***	0,247***	0,438***		0,391***		−0,282***
Minutes work/day		−0,928***						0,162**	
Conicity Index (cm)				0,469***	0,847***		0,366***		−0,295***
Waist Cincunmference (cm)					0,830***	0,276***	0,355***		−0,241**
WHtR (Kg/m^2^)							0,431***		−0,330***
% Fat Mass							0,459***		
Coronary Risk (points)								−0,315***	−0,244**

CI = Conicity Index; WC = Waist Cincumference; WHtR = Waist-To-Height Ratio; Rho = Spearman correlation coefficient; p = significance.

**p < 0,05 ***p < 0,001.

Table 3 shows the values obtained by multiple linear regression analysis between the demographic, occupational, anthropometric, leisure physical activity, coronary risk and physical health-related quality of life variables of the participants.

**Table 3:** Risk factors for coronary risk and the physical domain obtained by linear regression analysis

**Variable**	**Coronary risk (points)**				**Physical component (points)**			
	**β**	**t**	**IC**	**p**	**β**	**t**	**IC**	**p**
WHtR (cm)					−0,351	−4,785	−62,19/-25,85	< 0,001
Fat Mass Percentage	0,373	5,668	0,14/0,30	< 0,001				
Conicity Index	0,189	2,497	2,89/24,80	0,014				
Years of Service	0,248	3,59	0,06/0,22	< 0,001				
Minutes of Work Per Day	−0,133	−2,20	−0,004/-0,00	0,029				
Leisure Physical Activity level (mets)	−0,222	−3,63	−0,001/0,000	< 0,001				

WHtR = Waist-To-Height Ratio; β = Standardized score in standard deviation; t = value of the test statistic; CI = confidence interval, p = significance.

Multiple linear regression analysis identified fat percentage, conicity index, minutes of work per day, years of service and leisure-time physical activity levels as risk factors for coronary disease and identified waist-to-height ratio as a risk factor for physical component of health-related quality of life.

Analysis of the determination coefficients (R^2^) indicated that 41% of the variation in coronary risk, adjusted according to the sample, can be attributed to variations in fat percentage, conicity index, minutes of work per day, years of service and leisure-time physical activity levels. Analysis also revealed that 11.8% of the variation in physical health-related quality of life can be attributed to variations in waist-to-height ratio.

Analysis of the regression coefficients indicated that each increase of 0.22% in fat mass percentage, 13.84 in conicity index, 0.14 in years of service, 0.002 minutes of work per day in led to a one point increase in coronary risk.

Analysis further indicated that each decrease of 0.001 in MET of leisure-time physical activity led to a one point increase in coronary risk. Each one inch increase in the waist-to-height ratio contributed to a 44.02 reduction in physical health-related quality of life.

## Discussion

This study found associations between subjects’ fat percentage, conicity index, minutes of work per day, years of service, leisure-time physical activity levels, coronary risk and physical component of health-related quality of life.

Several studies involving public safety professionals from different backgrounds have identified years of police service as a risk factor for body composition changes and coronary disease. The results of the longitudinal study conducted by Boyce et al. [5] with the Charlotte Police Department indicated that years of police service contributed to increases in body composition values. They observed significant increases in body mass, fat percentage and fat mass after a decade of police work. Similarly, in a longitudinal study by Sörensen et al. [2] with Finnish police, there was an increase in body weight and waist circumference seen over 15 years of police work. In other words, the proportion of overweight individuals (IMC < 27) among the Finnish police officers was considerably lower in 1981 than in 1996 (29% and 51%, respectively); in addition, almost two thirds of the police officers (64%) had a waist circumference of > 94 cm, and more than a third (38%) had a waist circumference > 102 cm.

Beyond the time of service, Ferreira, Augusto Bonfin and [17] (2012) authors identified after multivariate analysis that the absence of weekly days off is also an important determinant of morbidity among military police of the city of Recife, Brazil. Another problem demonstrated in this study was the number of minutes of work per day. Mean minutes of work submitted by the majority was 809.15 minutes, which equates to over 12 hours of work. In research Minayo, Assisi and Oliveira [18], working hours from eight to 12 hours were considered by respondents as highly stressful. The military policemen said they work day and night, get 12 hours on the street with just one meal, work under pressure, having to stay alert and sleep less are factors that contribute to the deterioration of their quality of life and your health.

Similar to this study, Yasmin [19], Ghosh, Fitzgerald, Bose and Chaudhuri [20], Pitanga and Lessa [21] found conicity index to be a better indicator of coronary risk. Pitanga and Lessa [13] established a cutoff value of 1.25 for men for predicting cardiovascular risk. The average of the conicity index value in this study is near the recommended range.

Furthermore it was observed that most police officers showed a high percentage of fat. One explanation for this condition would be inadequate consumption of food, as many times the policemen have access only to convenience store or fast food, lack of awareness of a healthy lifestyle, besides the limited opportunities that these workers have to practice exercises physical.

Studies also suggest that anthropometric changes related to obesity represent a health problem among police officers [2,5,8,22] because excess body fat is a risk factor for cardiovascular diseases that affect occupational performance. Even in a study of subjects who are not police officers, Harvey et al. [23] reported that obese workers are prone to illness, absenteeism and early retirement when compared to non-obese workers.

The present study notes that most police officers have low leisure-time physical activity levels. This result may be related to the search for a second job. In a study with police officers, Minayo [24] concluded that because of low pay, some police officers have a second job to supplement their wages. This enhances the possibility of a relationship between low leisure-time physical activity levels and secondary employment in the population in question. Moreover, this practice can also interfere the rest and the family life [25]. Another factor that may be related to the low level of physical activity during leisure time is police work itself, which is considered an extremely stressful activity that causes a physical overload resulting from long working hours, night work schedules or for long periods at position orthostatic [26].

This study also showed a significant association between leisure-time physical activity levels and coronary risk. According to Barros and Santos [27], physical inactivity is considered a risk factor for coronary heart disease. They found that compared to regularly and moderately active people, inactive individuals are twice as likely to suffer a heart attack, independent of other risk factors.

The physical demands of police work are often inadequate for the maintenance of physical fitness; consequently, there is a low level of physical activity among police officers [28]. The fact that police officers are not involved in physically active professional tasks would not have adverse effects if they participated in more leisure-time activities and were not exposed to stressful or harmful situations.

Police work has been identified as a major source of psychological distress among police officers, as these professionals deal with acts violence, brutality and death in their daily lives [29-31].

These events, which are associated with long work hours and the possibility of being injured or killed, elevate stress-related symptoms in this population, compromising their quality of life, health, job demands, and relationships with peers and family [29]. Data obtained from Sector Statistics of the General Board of Health, from the Ambulatory Control of Attendances (CAAL) from Psychology in the year 2009 about the mental health of military police indicate that 70% have neurotic disorders and stress-related disorders, 18% have mood disorders and 5% have other mental and behavioral disorders [26].

In addition to psychological distress, physical illness can also affect police officers’ general quality of life [29,32]. Injuries by firearms and trauma were identified by civil and military police of Rio de Janeiro as the most common physical illnesses in their daily work [18].

A study by Chen et al. [29] showed that police officers with physical illnesses have lower mental health scores, as measured with the SF-12. This is consistent with a study by Surtees et al. [32], indicating that physical illness affects physical and mental health-related quality of life. This study identified waist-to-height ratio as a risk factor for physical health-related quality of life among military police officers.

Muniz and Soares conducted a mapping of victimization of police in Rio de Janeiro and observed that 93% of police officers were removed from the ostensible services to perform internal tasks due to physical disability. In 1997, 50.2% of the allowances to health care (LTS) and 42.8% of partial disabilities (IFP) were caused by trauma, and the LTS 5.6% and 16.9% of the IFP were to to psychiatric problems [33]. In both cases, Souza and Minayo [34] emphasize the risks and stress experienced at work. In research of Souza and Minayo [34] on morbidity and mortality linked to work of 4,518 military police of the city of Rio de Janeiro, deaths and injuries from all causes between the years 2000–2004, 56.1% were victimized during clearances, against 43.9% in service.

Physical preparation is extremely important for military police activities because of the adversity that police officers encounter in their daily lives. To effectively preserve public order and complete various job functions, police officers must possess a high level of fitness and good health. Military police training emphasizes physical fitness and health as part of its curriculum, which includes self-defense, police techniques and extracurricular activities. However, as these professionals enter the workforce in various military organizations and functions of the State, frequently there is neither continuity in fitness nor continued monitoring of health conditions (i.e., there is no promotion of the practice of physical, sports and leisure activities in the Military Police).

According to the note of Instruction nº 002/PM-3/95 of the Military Police of Santa Catarina, instructors are authorized to carry out physical activities twice a week. This note was ratified by the order nº 245 019/Cmdo/2003, which emphasizes that “chiefs and directors should encourage and provide subordinates to practice physical activity.” However, when practiced, the activity is usually limited to participating in a sport, which is performed without satisfying the technical criteria for physical health and fitness in the Military Police.

Therefore, it is observed that the concern with health, with the performance of these professionals and the quality of life of the related professional activity demonstrated in this study is similar to other regions of Brazil and other countries in different contexts [2,5,6,8,17,18,24,29],[31]. Thus, there is the need for the Military Police Department to adopt an institutional policy that allows police officers to practice regular physical activity in order to maintain and improve their physical fitness, health, job performance and quality of life. Moreover, it seems plausible that by adopting an institutional policy that allows for the practice of regular physical activity and access to health benefits may also help promote a more peaceful and respectful relationship with society.

This study has three basic limitations: 1) the results only represent a single battalion of military police in the region of Florianopolis-SC. However, it is necessary to collect data from other battalions in order to verify the characteristics and needs of the population in question; 2) The International Physical Activity Questionnaire (IPAQ), which was used to assess physical activity levels, is prone to recall bias because it depends on memory recollection; 3) The data of some variables were obtained only by participant reports, which can be prone to self-generated information bias.

Despite the limitations, this study provided information on the effect of demographic, occupational, anthropometric characteristics, level of physical activity during leisure time on coronary risk and quality of life of military police.

This study concludes that waist-to-height ratio is a risk factor for physical component of the health related quality of life. Furthermore, waist-to-height ratio, fat percentage, years of service and leisure-time physical activity levels are risk factors for coronary disease among military police officers. These results suggest that policies to promote physical activity, sport and leisure among military police officers should be encouraged.

These results further highlight the importance of constructing and validating specific instruments for this population. This may contribute to the production of more reliable data. Consequently, the results are more robust and may allow for more effective interventions. Further studies on this topic are needed.

## Competing interests

The authors declare that they have no competing interests.

## Authors’ contributions

FCS was responsible for identifying the research question, the design of the study, and data collection. FCS, SSSH, BAVA, and TLSC were responsible for the data collection and correcting the article. FCS and PJBGF contributed to the fine-tuning of the methodology and to correcting the article. RS was responsible for the review and revision of the manuscript. All authors helped to revise the manuscript and approved the final version.
